# Nutritional Intervention Reduces Dyslipidemia, Fasting Glucose and Blood Pressure in People Living with HIV/AIDS in Antiretroviral Therapy: A Randomized Clinical Trial Comparing Two Nutritional Interventions

**DOI:** 10.3390/nu12102970

**Published:** 2020-09-28

**Authors:** Erika Aparecida Silveira, Marianne Oliveira Falco, Annelisa Silva e Alves de Carvalho Santos, Matias Noll, Cesar de Oliveira

**Affiliations:** 1 Department of Epidemiology & Public Health, Institute of Epidemiology & Health Care, University College London, London WC1E 6BT, UK; 2 Postgraduate Program in Health Sciences, Faculty of Medicine, Federal University of Goiás, Goiânia 74605-050, Brazil; marianne.falco@gmail.com (M.O.F.); annelisa.nut@gmail.com (A.S.e.A.d.C.S.); 3 Department of Public Health, Instituto Federal Goiano, Ceres 76300-000, Brazil; matiasnoll@yahoo.com.br

**Keywords:** HIV, AIDS, antiretroviral therapy, dyslipidemia, cardiometabolic risk factors, dietary intervention, nutritional counseling, individualized dietary prescription

## Abstract

Antiretroviral therapy (ART) increases the risk of cardiometabolic diseases in people living with HIV/AIDS (PLWHA). However, there is a lack of evidence regarding the effectiveness of a nutritional intervention on several cardiometabolic parameters in this population. Therefore, this study aimed to evaluate the effectiveness of two nutritional interventions on several cardiometabolic parameters in PLWHA treated with ART. A parallel randomized clinical trial was performed with PLWHA treated with ART. The participants (*n* = 88) were divided into two intervention groups: (1) nutritional counseling (*n* = 44) and (2) individualized dietary prescription (*n* = 44). The follow-up period was 30 weeks. A reduction in low-density lipoprotein (LDL) was the primary outcome. Secondary outcome variables were reductions in total cholesterol (TC), triglycerides (TG), fasting plasma glucose (FPG), systolic and diastolic blood pressures (SBP and DBP, respectively), waist circumference (WC), body mass index (BMI), and increases in high-density lipoproteins (HDL). A multiple linear regression was used to analyze the effectiveness of the interventions, adjusted for sociodemographic, lifestyle, and clinical characteristics. Sixty-two PLWHA completed the trial (nutritional counseling, *n* = 32; individualized dietary prescription, *n* = 30). At follow-up, we observed in the nutritional counseling group significant reductions in SBP (*p* = 0.036) and DBP (*p* = 0.001). Significant reductions in FPG (*p* = 0.008) and DBP (*p* = 0.023) were found in the individualized dietary prescription group. In the fully adjusted models, significant reductions in LDL, SBP, DBP, and BMI were found in the individualized dietary prescription group. In conclusion, the two investigated nutritional interventions were effective in reducing some cardiometabolic risk factors in PLWHA. However, after adjustments for covariates, the individualized dietary prescription showed significant reductions in the primary outcome and, also, in more cardiometabolic risk factors than the nutritional counseling.

## 1. Introduction

Despite the immense benefits that antiretroviral therapy (ART) use has brought to people living with HIV/AIDS (PLWHA), there are some associated increased risks of dyslipidemia, hyperglycemia/diabetes, gastrointestinal symptoms, obesity, and hypertension, which contribute to a higher cardiometabolic risk in this population [1,2,3,4,5,6,7]. Other risk factors also corroborate for cardiometabolic diseases in PLWHA, such as smoking, excessive alcohol consumption, and physical inactivity [1,8,9,10,11,12].

Clinical treatment guidelines for PLWHA include prevention and treatment of cardiometabolic risk factors. However, most of the clinical recommendations are based on drug treatments [13]. Therefore, it is important to explore non-pharmacological treatments. Reducing cardiometabolic risk factors is essential in the treatment of PLWHA in ART, and nutritional interventions have an important role in the management of metabolic abnormalities [14].

There is little evidence on the effectiveness of nutritional treatment on metabolic abnormalities in PLWHA, specially dyslipidemia, fasting plasma glucose (FPG), blood pressure, body mass index (BMI), and waist circumference (WC) in PLWHA treated with ART [10,15,16,17,18,19,20]. In a meta-analysis that evaluated the effects of dietary interventions on HIV-associated dyslipidemia, most studies evaluated specific nutrient supplementation and only few studies evaluated dietary interventions, often combined with exercise programs [15]. With regard to dietary interventions, most studies followed recommendations from general guidelines [16,21,22], but none of the previous studies had analyzed and compared the effectiveness of different nutritional treatment approaches [15,23].

The type of nutritional approach with a difference in the number of dietitian consultations and prescription could influence the dietetic treatment and, consequently, reduce the cardiometabolic outcomes. According to the above concern, our study is based on the following research question: Could an individualized dietary prescription with one consultation per month be a more appropriate and effective approach in reducing cardiometabolic risk factors in PLWHA than nutritional counseling with fewer dietitian consultations? Therefore, it is important to analyze whether different nutritional approaches can lead to different cardiometabolic outcomes [24,25,26].

In this context, this study aims to investigate the effectiveness of two nutritional treatment approaches on cardiometabolic risk factors reduction in PLWHA treated with ART. The included cardiometabolic risk factors were dyslipidemia, FPG, blood pressure, BMI, and WC. We also investigated whether the effectiveness of these two interventions (nutritional counseling and individualized dietary prescription) could be modified by sociodemographic, lifestyle, and clinical characteristics.

## 2. Methods

### 2.1. Study Design

This study was an open controlled randomized clinical trial (RCT) with parallel intervention, nested within a major clinical cohort entitled Predictors of cardiovascular disease in PLWHA (PRECOR) [2]. The PRECOR study aimed to assess cardiovascular risk and metabolic abnormalities in PLWHA being monitored in a referral hospital for the care of infectious and parasitic diseases. The RCT was named PRECOR-NUT [6,7,27] and registered at ClinicalTrials.gov (NCT02180035). Data collection was performed in the outpatient clinic of the Infectious and Parasitic Diseases Service of the Clinical Hospital of the Federal University de Goiás, Goiânia, Brazil. This is a reference outpatient clinic in the treatment of HIV/AIDS in the State of Goiás.

Eligible individuals were HIV-infected adults aged 19 years or older treated with ART for at least 30 days attending the outpatient clinic at the time of recruitment. Exclusion criteria were pregnancy or lactation and diagnosis of any opportunistic disease in the last 2 months prior to enrollment in the major study.

Before starting baseline procedures, training and standardization of the entire data collection were conducted. The research team consisted of a cardiologist, physical educator, nutritionist, and anthropometrist. The training was very detailed, especially for nutritionists involved in the interventional procedures and for those responsible for the anthropometric measurements [28], to ensure high quality and uniformity of all procedures.

### 2.2. Baseline

Before randomization, at baseline, the nutritionist applied a standardized structured questionnaire covering clinical and lifestyle variables such as smoking status, alcohol consumption, and physical activity. After the first consultation with the nutritionist, the anthropometrist performed anthropometric measurements including of body weight, height, and waist circumference.

### 2.3. Enrollment and Randomization

The PRECOR cohort study consisted of 337 PLWHA. During routine care with the infectious disease physician at the Infectious and Parasitic Diseases Service, eligible individuals were referred to a consultation with the cardiologist who conducted a structured questionnaire covering sociodemographic and clinical questions and requested biochemical tests, i.e., lipid profile and fasting plasma glucose.

The eligible PRECOR participants were referred to the nutritionist, who invited them to participate in this study. Upon acceptance, the individuals signed to give informed consent and 88 participants were randomly allocated to one of the two intervention groups in a 1:1 ratio according to a random sequence generated by a randomization website. This study had two arms: a nutritional counseling group and a diet group. The interventions started with 88 PLWHA, 44 in each arm. The sample size estimate was performed based on the central limit theorem. According to this theorem, a sample with a size equal to or greater than 30 tends to present normality in the distribution of means and is also enough to find significant differences [29].

**Figure 1:**
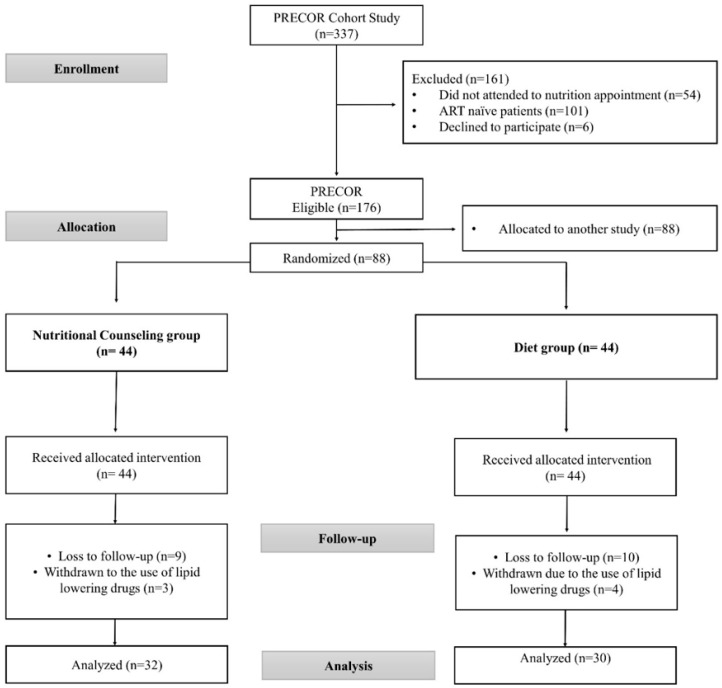
Flowchart of entry in clinical trial and follow-up of the participants in groups-nutritional counseling group and diet group. Completers’ analysis.

Out of the 337 PLWHA participating in the PRECOR cohort study, 176 patients were randomized to participate in the present study since 54 patients did not attended the first nutrition consultation, 101 were ART naïve patients, and 6 declined to participate. Out of the 176 randomized referred patients, 88 were allocated to another study (Figure 1).

### 2.4. Blinding

After randomization, patients’ appointments were scheduled on different days of the week to avoid contact between groups and prevent information exchange regarding the received intervention.

### 2.5. Intervention Protocols

There were two intervention groups: (1) nutritional counseling and (2) individualized dietary prescription. The individualized dietary prescription takes into account the energy and nutritional needs of each individual in addition to their biopsychosocial context. In both groups, nutritional care was provided by a trained nutritionist. The nutritional counseling group received nutritional guidance on promoting healthy eating using the “10 steps to healthy eating” folder, from the Brazilian Ministry of Health [30], that is part of the first edition of the Nutritional Guide for the Brazilian Population, which was the only version available at the time of the study.

The 10 healthy eating steps were as follows: (1) Make at least three meals (breakfast, lunch, and dinner) and two healthy snacks per day. Do not skip meals. (2) Include six portions of the cereal group (rice, corn, wheat, bread and pasta), tubers such as potatoes and roots such as cassava in meals. Give preference to whole grains and foods in their most natural form. (3) Eat at least three servings of vegetables daily as part of meals and three or more servings of fruit in desserts and snacks. (4) Eat beans with rice every day or at least five times a week. This Brazilian dish is a complete combination of proteins and good for health. (5) Consume three servings of milk and dairy products daily and a portion of meats, poultry, fish, or eggs. Removing the apparent fat from meat and poultry skin prior to preparation makes these foods healthier! (6) Consume a maximum of one portion per day of vegetable oils, olive oil, butter, or margarine. Watch for food labels and choose those with the lowest amounts of trans-fats. (7) Avoid soft drinks and processed juices, cakes, sweet and stuffed biscuits, sweet desserts, and other treats as a rule for feeding. (8) Decrease the amount of salt in the food and remove the saltshaker from the table. Avoid consuming high-sodium (processed) foods such as hamburger, sausage, ham, snacks, canned vegetables, soups, and ready-to-eat sauces and seasonings. (9) Drink at least two liters (six to eight glasses) of water a day. Give preference to water consumption during meal breaks. (10) Make your life healthier. Practice at least 30 min of physical activity every day and avoid alcoholic beverages and smoking. Keep your weight within healthy limits.

The individualized dietary prescription group received a healthy eating plan which contained an individualized menu with mealtimes and a list of equivalent foods for each food group (bread/biscuit, milk/cheese, fruits, beans, vegetables, meat, oil/butter, sugar/candies) quantified in standard serving sizes using common kitchen measurements [31]. The healthy eating plan prescription took into consideration the socioeconomic status, lifestyle, and eating habits of each study participant. The nutritionists calculated individual energy and protein requirements [32] as well as the resting energy expenditure [33]. Adjusted weight was used for obese and underweight participants [34]. The daily macronutrient distribution range according to the total energy value was 55% to 60% carbohydrate, 25% to 30% fat, 15% protein, along with 20 to 30 g of dietary fiber [13,35]. The diet group received instructions to not consume foods containing trans-fat [13,31,35]. In addition, this group was instructed to prepare meals with less fat and sugar, prioritizing baking, grilling, and steaming while avoiding frying. Patients in both groups received nutritional guidelines as informative standard forms in case of hypercholesterolemia, hypertriglyceridemia, hypertension, and diabetes, independently of the intervention group to which they were allocated [31].

### 2.6. Follow-Up

The nutritional treatment and follow-up visits were specific to each group. Both groups were followed for approximately 30 weeks. After randomization, return visits were scheduled at week 14–15 for the nutritional counseling group and at every four or five weeks for the diet group. This difference in the number of consultations is part of the intervention style which we are testing.

During each follow-up visit, food intake, body weight, and waist circumference were assessed. The compliance to dietary intervention was carried out by assiduity in return visits and by the dietitian’s perception during consultations. The compliance to the dietary intervention was evaluated by one registered dietitian who received training to assess participants’ motivation and standardize the treatment protocols and approaches. The decision to have only one dietitian was a strategy to provide and ensure standardized treatment in both groups. Therefore, the same nutritionist provided patient care throughout the study, strengthening the professional–patient relationship.

### 2.7. Study Variables

The study variables analyzed were sociodemographic (sex, age, skin color, marital status, income, and educational level); lifestyle (smoking status, alcohol consumption, and physical inactivity); clinical (family history of cardiovascular disease, viral load, time of ART use, class of antiretroviral drug, and blood pressure); anthropometric (body weight, height, WC, and BMI) and biochemical (lipid profile and fasting plasma glucose).

#### 2.7.1. Sociodemographic Variables

Income was collected according to patients’ monthly income and grouped into quartiles in Brazilian real (BRL-R$): 1st quartile, minimum income up to R$509.00; 2nd quartile, income from R$510.00 to R$699.00; 3rd quartile income from R$700.00 to R$1199.00, and 4th quartile, income equal to or greater than R$1200.00. The average exchange rate during the study period was 1 USD = 3.57 BRL. Schooling years were grouped into four categories: up to 4 years; 5 to 8 years; 9 to 11 years; and 12 or more years of study.

#### 2.7.2. Lifestyle Variables

Smokers were considered those who smoked or stopped smoking less than six months prior to the study, while non-smokers and ex-smokers were those who stopped smoking for more than 6 months prior to the study [36].

Alcohol consumption was investigated according to the type of beverage, frequency, and amount (doses, bottles, or glasses) consumed in the week prior to the first study interview [37]. The amount of alcoholic beverages consumed was converted to grams of ethanol per day.

Physical activity was assessed using the short version of the International Physical Activity Questionnaire (IPAQ) [38,39]. Those with no or low levels of physical activity were classified as sedentary, i.e., a score less than 600 MET-min/week [38,39].

#### 2.7.3. Clinical Variables

Viral load values (copies/mL) were classified as <50 (undetectable viral load) and ≥50 [40,41]. Antiretrovirals drugs were categorized into nucleoside-analogue reverse transcriptase inhibitors (NRTI), non-nucleoside reverse transcriptase inhibitors (NNRTI), and protease inhibitors (PI).

The Welch Allyn/Tycos aneroid sphygmomanometer was used to measure arterial blood pressure. The participant was asked to sit with their legs uncrossed, back supported, and their arm positioned so that the upper part of the cuff was at the height of the midpoint of the sternum. Three successive measurements were taken with a one-minute interval between measurements. The first measurement was performed after five minutes of rest [42,43,44].

#### 2.7.4. Anthropometric Variables

The anthropometric variables (body weight, height, and WC) were measured according to a standardized protocol [45]. The Tanita BC558-Ironman digital scale with a capacity of 150 kg and an accuracy of 100 g was used to measure body weight. For the height measurement, we used a tape affixed to a wall with an accuracy of 0.1 cm. The BMI value was calculated by dividing body weight in kilograms by the square of height in meters.

#### 2.7.5. Biochemical Variables

For the biochemical tests, study participants were asked to fast for 12 h and avoid consumption of alcohol for three days before blood collection. The total lipid profile values were obtained by an automated enzymatic method following established techniques [46,47]. LDL was calculated using the Friedewald et al. equation [48] if triglycerides < 400 mg/dL. LDL was defined as the primary outcome. The fasting plasma glucose was obtained through biochemical analysis in peripheral blood.

### 2.8. Ethical Considerations

This RCT was conducted according to the ethical standards established in the Declaration of Helsinki. The Ethics Committee on Medical, Human and Animal Research of the Federal University of Goiás Clinical Hospital approved the study protocol (no. 163/2009).

### 2.9. Statistical Analysis

The Shapiro–Wilk test was used to assess the normality of the continuous data distribution. Pearson’s Chi-squared and Fisher’s exact tests were used in the bivariate analyses. The paired and unpaired Student’s *t*-test, Wilcoxon test, and Mann–Whitney test (nonparametric data) were employed for the continuous variables. McNemar’s test was used to compare paired categorical variables at baseline and at the end of follow-up in each intervention group.

The primary outcome was LDL reduction. Secondary outcomes were reductions in TC, TG, FPG, SBP, DBP, WC, and BMI. We also analyzed the increase in HDL as an outcome. The effectiveness of each treatment on the outcome variables was calculated by the difference between baseline and final follow-up values for each intervention group [49].

We performed linear regression between the outcomes and the independent variables. Those associations that showed a *p*-value smaller than or equal to 0.20 at this stage of analysis were included in the multiple linear regression analysis. Lastly, only those variables with a *p*-value smaller than or equal to 0.05 were kept in the final multiple linear regression models.

The database was structured in EpiData version 3.0 with double entry. All analyzes were performed using the Stata 12^®^ statistical program (Stata Corp, College Station, TX, USA).

## 3. Results

Out of 176 individuals referred to this study from the 337 PLWHA participating in the PRECOR cohort study, 88 were allocated to another clinical trial study. Therefore, the present study comprised 88 participants, with 44 allocated to the nutritional counseling group and 44 to the diet group. Losses of follow-up and exclusions with reasons are displayed in Figure 1. Sixty-two patients successfully completed the study and were analyzed at the end of follow-up, after approximately 30 weeks, with 32 participants being in the nutritional counseling group and 30 participants in the individualized dietary prescription group.

The main sociodemographic characteristics of the included PLWHA were 67.74% men, 48.39% had brown/black skin, 54.84% had a monthly income higher than R$700.00, and 59.68% had nine or more years of education (Table 1). Regarding lifestyle, 61.29% did not smoke, 51.61% consumed alcohol, and 58.06% were physically inactive. The ART use time was over three years in 33.33% of the participants, and 70.40% used it for over six months. After randomization, both intervention groups were similar except in relation to their FPG (Table 1). NRTIs are not presented in the tables because all PLWHA used these drugs.

Comparing the data from baseline to the end of follow-up within each group, there was a statistically significant reduction in DBP for both groups. The individualized dietary prescription group had a significant reduction in FPG (*p* = 0.008). The nutritional counseling group had statistically significant decreases in SBP (*p* = 0.036) and DBP (*p* < 0.001) (Table 2).

In both groups, a reduction in LDL, TC TG, FPG, SBP, DBP, and WC, and an increase in HDL values between baseline and the end of follow-up were observed. Clinically, the reduction in TC, LDL, TG, FPG, and WC were more expressive in the individualized dietary prescription group. Regarding WC, in the individualized dietary prescription group, there was a reduction of 1.74 cm, while in the nutritional counseling group, there was a reduction of only 0.30 cm. With respect to BMI, it was reduced only in the individualized dietary prescription group (−0.36 kg/m^2^), while there was an increase in the nutritional counseling group (0.22 kg/m^2^) (Table 3).

In the nutritional counseling group, the variables included in the multiple linear regression analysis for each outcome were as follows: income for TC; sex, income, smoking, ART time use, and NNRTI for LDL; income, education, and smoking for HDL; sex and age for TG; age, skin color, ART time use, NNRTI, and IP for FPG; smoking and time of ART use for SBP; skin color and income for WC (*p*-value < 0.20). After multiple linear regression, HDL was reduced by 4.6 mg/dL (*p* = 0.013) among those patients in the 4th quartile of income, TG increased to 12.57 mg/dL (*p* = 0.019) in those aged 40 years or more and WC also increased to 1.48 cm (*p* = 0.036) in those with brown/black skin. In this group, SBP was reduced by 0.27 mmHg in those using ART for more than a year (*p* = 0.024). Despite the four significant *p*-values in both intervention groups, the primary outcome LDL was significant only in the diet group. Both interventions showed reductions in cardiometabolic risk factors for HIV patients (Table 4).

In the individualized dietary prescription group, the following variables were included in the multiple linear regression analysis: ART time use, family history of cardiovascular disease, and alcohol consumption for TC; skin color, family history of cardiovascular disease for LDL; income and ART time use for HDL; skin color, age, income, smoking, and ART time use for TG; income, education, and NNRTI for FPG; skin color, physical inactivity, alcohol consumption, and IP for DBP; sex, skin color, and consumption of alcoholic beverages for SBP; sex for WC; and ART time use for BMI (*p*-value < 0.20). There were statistically significant reductions in the following: LDL (−24.76 mg/dL) in those with brown/black skin, SBP (−12.20 mg/dL) in women, DBP (−9.85 mmHg) in those using IP and BMI (−0.03 kg/m^2^) between those who used ART for more than three years (Table 4).

## 4. Discussion

To the best of the authors’ knowledge, this is the first clinical trial with PLWHA treated with ART that demonstrated the effectiveness of nutritional intervention on reducing several cardiometabolic risk factors in both nutritional intervention groups, i.e., nutritional counseling and individualized dietary prescription. In the nutritional counseling group, significant reductions were observed for systolic and diastolic blood pressure, while in the individualized dietary prescription group, significant reductions were observed for diastolic blood pressure and fasting plasma glucose. In the multivariate model with subgroup analysis, we found that sociodemographic, lifestyle, and clinical parameters can influence the effectiveness of some outcomes, an important contribution of this RCT. More effective reductions were found in the individualized dietary prescription group compared to the nutritional counseling group. However, the nutritional counseling could also be applied in HIV/AIDS ambulatory care settings if there were not enough dietitians to prescribe individualized diets.

In both groups, the nutritional treatment significantly decreased DBP levels. In the nutritional counseling group, we observed SBP decreases, while in the individualized dietary prescription group, the reduction was relevant but of marginal significance. Only another RCT found reductions in SBP [50], however, it included nutritional intervention and physical activity. RCTs with nutritional counseling found no reductions in DBP and SBP [22]. In our study, the average reduction in DBP was up to 5.2 mmHg and was 4.4 mmHg for SBP. These reductions may have an important clinical role in controlling blood pressure in PLWHA, especially considering that the average blood pressure in both groups at baseline was normotensive.

Nutritional treatment for PLWHA may have an important preventive role in hypertension, especially considering that blood pressure increases significantly during 96 weeks of antiretroviral use [51]. Some evidence recommends the DASH diet or the Mediterranean diet for the management of hypertension [52]; however, this type of diet is not part of the eating habits of most PLWHA, since two-thirds of PLWHA live in the Americas and sub-Saharan Africa [53]. However, the present study provided a reduction in DBP and SBP with nutrition advice or individualized dietary prescription, respecting regional eating habits.

Fasting plasma glucose showed a statistically significant reduction in the individualized dietary prescription group with an average reduction of 3.8 mg/dL. The few studies that evaluated FPG in PLWHA treated with ART observed no reduction in glycemic parameters when prescribing dietary interventions [54] and nutritional counseling [50]. Changes in glycemic profile are risk factors for cardiometabolic diseases [55]; this is the first study evaluating this parameter with nutritional intervention in PLWHA treated with ART [56]. However, this result for FPG could be attributed to differences in baseline values and not due to the intervention.

In this RCT, there was an average reduction in WC close to being significant (*p* = 0.07). Similar results were observed in another RCT of 0.9 cm at the end of follow-up [10]. The few studies that found a reduction in WC were conducted with overweight PLWHA (BMI > 25 kg/m²) [50,54] while in the present study, the BMI at baseline was within the normal range. In the individualized dietary prescription group, there was a reduction of 1.74 cm in WC, in contrast to an observational study that found an increase in WC in PLWHA treated with ART [51]. This reduction observed in this RCT may be important in the long term to prevent abdominal obesity. BMI remained stable in both groups. Another RCT with nutritional counseling observed a significant reduction in BMI in the intervention group whilst this remained stable in the control [22].

One aspect that deserves special attention in RCTs is the clinical relevance of the results regardless of *p*-values [57]. In the present study, although some outcomes did not show statistically significant reductions, they are worth mentioning. For example, the reductions in TC (−9.4 and −4.3 mg/dL), LDL (−10.0 and −2.9 mg/dL) and TG (−27.5 and −21.4 mg/dL) and increases in HDL (1.8 and 2.8 mg/dL) observed in the individualized dietary prescription and nutritional counseling group, respectively. Overall, in the individualized dietary prescription group, the reductions were slightly more expressive, so the prescription of an individualized food plan may contribute to improvements in LDL, TC, and HDL levels in PLWHA treated with ART.

An important feature of this RCT was the application of multiple linear regression analysis with dummy variables, allowing the identification of changes in the effectiveness of treatment in groups of patients with distinct characteristics. In the individualized dietary prescription group, an even greater effectiveness of treatment with significant reductions were observed in some patients: LDL (−24.8 mg/dL) among those with brown/black skin color, BMI in those treated with ART longer than three years, and SBP (−12.2 mmHg) in females, while for the other patients, there was no significant reduction in these cardiometabolic parameters. The DBP that already had a significant reduction of 3.4 mmHg was further reduced in those treated with PI when compared to other antiretroviral drugs, reaching −9.8 mmHg.

Some of our results showed significant worsening in the outcomes due to intervention in the nutritional counseling group. For example, there were increases in TG values (12.6 mg/dL) among those aged 40 and older, an increase in WC (1.5 cm) for those with brown/black skin color, reduction in HDL (−4.6 mg/dL) for patients in the 4th quartile of income and, finally, a reduction in SBP of only 0.3 mmHg among those treated with ART longer than one year, which is considerably lower than other patients who had a reduction of 5.2 mmHg. This type of statistical approach allowed us to better understand the clinical outcomes based on the fact that participants’ individual characteristics may increase or even reduce the effectiveness of the intervention. Therefore, the analytical approach adopted in this study that accounted for how subgroups influence on the outcomes is a breakthrough not only in the RCT field but also in therapeutic approaches in clinical practice.

## 5. Limitations and Strengths

One possible limitation of this study was the lack of double-blindness, due to the kind of treatment approach [58]. For all types of behavioral interventions, such as physical activity and nutritional and psychological treatments, it is difficult to conduct a blind study because of the nature of the intervention, i.e., the participants know what is going on [58]. However, we tried to minimize such a limitation by using some strategies to prevent information exchange between groups, such as avoiding the contact between the intervention groups through different appointment schedules. Another potential limitation of our study could be attributed to the lack of a control group with no intervention. However, such a control group would not be approved by the ethics committee. The follow-up losses of both groups are similar and approximately 20%, which is expected in clinical trials.

Compliance to nutritional treatment is a subjective aspect difficult to analyze. Therefore, in our study, having only one dietitian with expertise was a good approach due to its good quality. In nutritional intervention studies, the dimension of adherence to dietary treatment is complex, and there is no method or instrument that could be applied properly to all the objectives and studies [59].

Regarding the clinical relevance [57] of our findings, we were able to highlight that an individualized dietary prescription could be a more appropriate and effective approach in reducing cardiometabolic risk factors in PLWHA than only nutritional counseling. In other words, in an individualized dietary prescription, the biochemical individuality could be taken into consideration. We recommend that future RCTs are developed on this topic and with subgroup analyses, such as the one carried out in the present study, since it is important to identify whether other variables can interfere with or modify the effectiveness of interventions.

It is known that, globally, the ambulatory care settings for HIV/AIDS in most cases do not include a registered dietitian, and nurses or physicians usually give a brief general nutritional orientation, if given. Some ambulatory care units just have one nutritionist to attend many patients. Therefore, if a general orientation, such as the one we had in one of the arms of our intervention, provides similar results to the diet prescription arm, this finding could be used to justify its use to help more patients. We showed that the general orientation had good results, but the individualized diet prescription demonstrated better results in reducing cardiometabolic risk factors. Our results are relevant in highlighting the relevance of nutritional treatments to reduce cardiometabolic risk factors. As the compliance was not different between the groups, the results demonstrate the effectiveness of nutritional intervention.

## 6. Conclusions

This RCT demonstrated the effectiveness of both nutritional interventions in reducing some cardiometabolic risk factors. However, the prescription of an individualized dietary plan was clinically more effective in reducing several cardiometabolic risk factors in PLWHA treated with ART, particularly DBP, FPG, TC, HDL, and the primary outcome LDL.

## Figures and Tables

**Table 1:** Sociodemographic, clinical, and cardiometabolic data at baseline for people living with HIV/AIDS (PLWHA) according to their allocated groups.

Variables	*n* (%) Total = 62	Nutritional Counseling Group *n* (%)	Diet Group *n* (%)	*p*-Value
**Sex**				0.861 *
Male	42 (67.74)	22 (52.38)	20 (47.62)	
Female	20 (32.26)	10 (50.00)	10 (50.00)	
**Age groups**				0.821 ^†^
≤29	11 (17.74)	6 (54.55)	5 (45.45)	
30–39	21 (33.87)	12 (57.14)	9 (42.86)	
40–49	21 (33.87)	9 (42.86)	12 (57.14)	
50 or above	9 (14.52)	5 (55.56)	4 (44.44)	
**Skin Color**				0.806 *
White	32 (51.61)	17 (53.13)	15 (46.88)	
Brown/black	30 (48.39)	15 (50.00)	15 (50.00)	
**Marital status**				0.355 ^†^
Single	32 (51.61)	17 (53.13)	15 (46.88)	
Married	14 (22.58)	5 (35.71)	9 (64.29)	
Widower/ divorced	16 (25.81)	10 (62.50)	6 (37.50)	
**Income quartiles**				0.602 ^†^
1st (poorest)	13 (20.97)	5 (38.46)	8 (61.54)	
2nd	15 (24.19)	9 (60.00)	6 (40.00)	
3rd	13 (20.97)	8 (61.54)	5 (38.46)	
4th (richest)	21 (33.87)	10 (47.62)	11 (52.38)	
**Education, schooling years**				0.526 ^†^
≤4 years	8 (12.90)	4 (50.00)	4 (50.00)	
5–8 years	17 (27.42)	8 (47.06)	9 (52.94)	
9–11 years	20 (32.26)	13 (65.00)	7 (35.00)	
>11 years	17 (27.42)	7 (41.18)	10 (58.82)	
**Smoking status**				0.980 *
Yes	12 (19.35)	6 (50.00)	6 (50.00)	
No	38 (61.29)	20 (52.63)	18 (47.37)	
Ex-smoker	12 (19.35)	6 (50.00)	6 (50.00)	
**Alcohol consumption**				0.450 *
Yes	32 (51.61)	18 (56.25)	14 (43.75)	
No	30 (48.39)	14 (46.67)	16 (53.33)	
**Sedentary behavior**				0.065 *
Yes	36 (58.06)	15 (41.67)	21 (58.33)	
No	26 (41.94)	17 (65.38)	9 (34.62)	
**Family History of Cardiovascular Disease**				0.230 ^†^
Yes	2 (3.23)	-	2 (100.00)	
No	60 (96.77)	32 (53.33)	28 (46.67)	
**Viral load**				0.110 ^†^
<50 copies/mL	47 (79.66)	22 (46.81)	25 (53.19)	
≥50 copies/mL	12 (20.34)	9 (75.00)	3 (25.00)	
**ART usage time**				0.135 ^†^
≤0.5 years	16 (29.63)	12 (75.00)	4 (25.00)	
0.5–1 year	5 (9.26)	4 (80.00)	1 (20.00)	
1–3 years	15 (27.78)	9 (60.00)	6 (40.00)	
>3 years	18 (33.33)	6 (33.33)	12 (66.67)	
**NNRTI**				0.204 *
Yes	43 (71.67)	20 (46.51)	23 (53.49)	
No	17 (28.33)	11 (64.71)	6 (35.29)	
**Protease Inhibitor**				0.050 ^†^
Yes	18 (30.00)	13 (72.22)	5 (27.78)	
No	42 (70.00)	18 (42.86)	24 (57.14)	
		**Average ± SD**	**Average ± SD**	
**Waist Circumference**	62	84.65 ± 9.39	88.02 ± 10.03	0.177 ^‡^
BMI (kg/m^2^)	62	23.81 ± 3.55	25.05 ± 3.90	0.197 ^‡^
Total cholesterol mg/dL	62	178.34 ± 47.59	197.80 ± 43.62	0.099 ^‡^
LDL-C (mg/dL)	62	103.72 ± 37.79	119.10 ± 38.78	0.122 ^‡^
HDL-C (mg/dL)	62	40.72 ±10.13	44.00 ± 12.26	0.287 ^‡^
Triglyceride (mg/dL)	62	167.88 ± 71.64	187.97 ± 90.33	0.464 ^§^
Fasting plasma glucose (mg/dL)	62	83.53 ± 7.73	89.80 ± 7.96	0.003 ^§^
Systolic pressure (mmHg)	62	117.53 ± 13.17	119.40 ± 13.77	0.587 ^‡^
Diastolic pressure (mmHg)	62	82.13 ± 8.43	80.57 ± 9.78	0.503 ^‡^

Values are *n* (%) or average ± SD. * Values obtained by Pearson X^2^ test. ^†^ Values obtained by Fischer Exact Test. ^‡^ Values obtained by Student’s *t*-test for independent variables. ^§^ Values obtained by Mann–Whitney U-test for independent non-parametric variables. PLWHA: People living with HIV/AIDS; ART: antiretroviral therapy; BMI: body mass index; HDL-C: high-density lipoprotein; LDL-C: low-density lipoprotein; NNRTI: non-nucleoside reverse transcriptase inhibitors; SD: standard deviation.

**Table 2:** Cardiometabolic variables: comparison of baseline data with the end of follow-up for each group of the randomized clinical trial.

Variables	Nutritional Counseling Group				Diet Group			
	*n*	Baseline	Follow-up	*p*-Value *	*n*	Baseline	Follow-up	*p*-Value *
Dyslipidemia	32	31 (96.88)	21 (65.63)	0.002 ^†^	30	29 (96.67)	22 (73.33)	0.039 ^†^
Isolated hypercholesterolemia ^a^	32	4 (12.50)	2 (6.25)	0.500 ^†^	28	3 (10.70)	3 (10.70)	1.000 ^†^
Hypertriglyceridemia	32	18 (56.25)	11 (34.38)	0.092 ^†^	30	20 (66.67)	12 (40.00)	0.032 ^†^
		Average ± SD				Average ± SD		
Total cholesterol (mg/dL)	32	178.34 ± 47.59	174.00 ± 43.88	0.423	30	197.80 ± 43.62	188.40 ± 37.32	0.099
LDL (mg/dL) ^#^	32	103.72 ± 37.79	100.84 ± 34.35	0.479	28	119.57 ± 39.41	109.54 ± 40.81	0.143
HDL (mg/dL)	32	40.72 ± 10.13	43.56 ± 10.06	0.095	30	44.00 ± 12.26	45.80 ± 14.31	0.266 ^‡^
Triglyceride (mg/dL)	32	167.88 ± 71.64	146.44 ± 69.21	0.112	30	187.97 ± 90.33	160.43 ± 91.28	0.089 ^‡^
Fasting plasma glucose (mg/dL)	32	83.53 ± 7.73	81.91 ± 8.37	0.275	30	90.14 ± 7.88	86.38 ± 9.72	0.008 ^‡^
Systolic pressure (mmHg)	32	117.53 ± 13.17	113.34 ± 10.94	0.036	30	119.40 ± 13.77	115.03 ± 9.39	0.066
Diastolic pressure (mmHg)	32	82.13 ± 8.43	76.94 ± 7.73	0.0009	30	80.57 ± 9.78	77.20 ± 7.10	0.023
Waist circumference (cm)	32	84.65 ± 9.39	84.42 ± 9.38	0.774	26 ^§^	88.77 ± 10.53	87.03 ± 9.99	0.068
BMI (kg/m^2^)	32	23.81 ± 3.55	24.03 ± 3.72	0.617	26 ^§^	25.14 ± 4.12	24.78 ± 4.09	0.151

Values are *n* (%) or average ± SD. ^a^ PLWHA without other associated dyslipidemias. * Values obtained by Student’s *t*-test for paired variables. ^†^ Values obtained by McNemar’s test for paired and categorical variables. ^‡^ Values obtained by Wilcoxon test for non-parametric paired variables. ^§^ Four PLWHA did not follow up to anthropometry. BMI: body mass index; HDL: high-density lipoprotein; LDL: low-density lipoprotein; SD: standard deviation. ^#^ The two missing individuals in the LDL group occurred due to missing blood assays in the laboratory.

**Table 3:** Effectiveness of each nutritional treatment on cardiometabolic variables.

Variables	*n*	Nutritional Counseling Group * Average ± SD	Diet Group * Average ± SD
Total cholesterol (mg/dL)	62	−4.34 ± 30.25	−9.40 ± 30.22
LDL (mg/dL)	60	−2.88 ± 22.70	−10.04 ± 35.18
HDL (mg/dL)	62	2.84 ± 9.34	1.80 ± 8.22
Triglyceride (mg/dL)	58	−21.44 ± 74.16	−27.53 ± 80.43
Fasting plasma Glucose(mg/dL)	58	−1.63 ± 8.27	−3.76 ± 8.35
Systolic pressure (mmHg)	62	−4.19 ± 10.80	−4.37 ± 12.52
Diastolic pressure (mmHg)	62	−5.19 ± 7.95	−3.37 ± 7.61
Waist circumference (cm)	58	−0.28 ± 4.52	−1.74 ± 4.65
BMI (kg/m²)	58	0.22 ± 2.41	−0.36 ± 1.22

* Values refer to the average difference of the results between the baseline and the end of follow-up. Values are average ± standard deviation (SD). Negative values represent a decrease and positive values represent an increase. BMI, body mass index; HDL, high-density lipoprotein; LDL, low-density lipoprotein; SD: standard deviation.

**Table 4:** Multiple linear regression of the effectiveness of each nutritional treatment on cardiometabolic outcomes.

Variables	Nutritional Counseling Group					Diet Group				
	A	Adjusted β * (95% CI)	Effectiveness	R²	*p*-Value	α	Adjusted β * (95% CI)	Effectiveness ^†^	R²	*p*-Value
Total cholesterol (mg/dL)	-	-	-	-	-	-	-	-	-	-
LDL (mg/dL)	-	-	-	-	-	2.74	−27.50 (−53.13 to −1.87) brown/black skin	−24.76	0.158	0.036
HDL (mg/dL)	8.34	−12.94 (−22.93 to −2.93) income 4° quartile	−4.6	0.206	0.013	-	-	-	-	-
Triglyceride (mg/dL)	−47.89	60.46 (10.44–110.48) Age > 40 years	12.57	0.169	0.019	-	-	-	-	-
Fasting plasma glucose (mg/dL)	-	-	-	-	-	-	-	-	-	-
Systolic pressure (mmHg)	−8.75	8.48 (1.19–15.77) >1 year of ART	−0.27	0.163	0.024	−0.45	−11.75 (−20.78 to −2.72) female	−12.2	0.202	0.013
Diastolic pressure (mmHg)	-	-	-	-	-	−17.68	7.83 (0.58–15.18) IP	−9.85	0.154	0.035
Waist circumference (cm)	−1.83	3.31 (0.22–6.40) brown/black skin	1.48	0.138	0.036		-		-	-
BMI (kg/m²)		-		-	-	−1.18	1.15 (0.09–2.22) >3 years of ART	−0.03	0.238	0.035

The values were obtained by a multiple lineal regression analysis. * Values were adjusted by sex, age, income, smoking, alcohol consumption, inactivity, family history of cardiovascular disease, ART usage time, protease inhibitor, non-nucleoside reverse transcriptase inhibitors/and nucleoside reverse transcriptase inhibitors. Blank spaces mean that none of the adjusted variables modified the effectiveness in cardiometabolic outcomes. ^†^ Effectiveness: difference among the values of each outcome variable between the baseline and the end of the follow-up. BMI, body mass index; ART, antiretroviral therapy; HDL, high-density lipoprotein; LDL, low-density lipoprotein; PI, protease inhibitor.

## Declaration

The lead author affirms that this manuscript is an honest, accurate, and transparent account of the study being reported. The reporting of this work is compliant with CONSORT guidelines. The lead author affirms that no important aspects of the study have been omitted and that any discrepancies from the study as planned (ClinicalTrials.gov NCT02180035) have been explained.
